# Low Bacterial Co-infection Invalidates the Early Use of Non-anti-*Mycoplasma pneumoniae* Antibiotics in Pediatric Refractory *Mycoplasma pneumoniae* Pneumonia Patients

**DOI:** 10.3389/fped.2018.00296

**Published:** 2018-10-26

**Authors:** Jin-Rong Liu, Jie Lu, Fang Dong, Hui-Min Li, Hui Liu, Xiao-Lei Tang, Yong-Li Guo, Shun-Ying Zhao

**Affiliations:** ^1^Department 2 of Respiratory Medicine, National Center for Children's Health, Beijing Children's Hospital, Capital Medical University, Beijing, China; ^2^Key Laboratory of Major Diseases in Children, Beijing Key Laboratory for Pediatric Diseases of Otolaryngology, Head and Neck Surgery, National Center for Children's Health, Beijing Pediatric Research Institute, Beijing Children's Hospital, Capital Medical University, Beijing, China; ^3^Biobank for Clinical Data and Samples in Pediatric, National Center for Children's Health, Beijing Pediatric Research Institute, Beijing Children's Hospital, Capital Medical University, Beijing, China; ^4^Department of Laboratory Medicine, National Center for Children's Health, Beijing Children's Hospital, Capital Medical University, Beijing, China

**Keywords:** bacterial infection, refractory, *Mycoplasma pneumoniae* pneumonia (MPP), NGS (next generation sequencing), antibiotics–rational therapy

## Abstract

**Background:** Childhood refractory mycoplasma pneumoniae (MP) pneumonia (RMPP) is a lung disease with elevated level of C-reactive protein and severe clinical and radiological deterioration. Whether bacterial co-infection contributes to disease of RMPP and whether inclusion of non-anti-MP antibiotics in treatment regimen would benefit RMPP patients remains elusive.

**Methods:** We retrospectively reviewed the medical records of 675 RMPP children. Traditional bacterial culture and next generation sequencing (NGS) were used to detect bacteria in bronchoalveolar lavage fluid in all the 675 patients and 18 patients respectively. Antibiotics used and clinical outcomes were analyzed along with other clinical measurements.

**Results:** Positive bacterial cultures were only found in 18 out of 675 cases (2.67%) and NGS analyses of another 18 cases did not revealed positive bacterial infection, which were consistent with the results of bacterial cultures. Non-anti-MP antibiotics were utilized in 630 cases (93.33%), even last-line antibiotics, such as glycopeptides or carbapenems, were frequently used.

**Conclusion:** Bacterial co-infection in RMPP was rare and non-anti-MP antibiotics didn't show any efficacy for early treatment of RMPP patients, which may provide a rationale for restricting the use of non-anti-MP antibiotics in RMPP patients and preventing antibiotic resistance globally.

## Introduction

*Mycoplasma pneumoniae* (MP) pneumonia (MPP) in children is a significant public health problem. Recently, more and more refractory or severe cases of MPP have been reported worldwide, especially in Asia (1–5). Most refractory MPP (RMPP) patients received treatments of macrolides followed by tetracyclines or quinolones. The RMPP patients are commonly associated with prolonged fever, high level of C-reactive protein (CRP) in peripheral blood, airway hypersecretion in bronchoscopy, and high-density consolidation in chest imaging (3–6), among which CRP is commonly used as an indicator of the presence of inflammation and infection especially bacterial infection (7–11).

In clinic CRP levels higher than 40 mg/L (sometimes higher than 160 mg/L, normal range < 8 mg/L) would prompt clinicians to consider potential bacterial co-infection and prescribe both non-anti-MP and anti-MP antibiotics to RMPP patients. However, subsequent bacteria culture rarely revealed the presence of bacterial co-infection and data in large population are scarce in RMPP patients. Thus, whether it is necessary to combine non-anti-MP antibiotics along with anti-MP antibiotics for children RMPP patients with CRP more than 40 mg/L needs to be further defined. Given that the pathogenesis of RMPP is mainly attributed to excessive cell-mediated immunity and cytokine responses against the pathogens (12, 13), it is plausible that the high level of CRP and other signs in RMPP patients may be caused by either excessive immune response or a combination of immune response and bacterial co-infection, the answer of which will be critical to guide rational therapy and prevent antibiotic misuse or overuse.

Here, we studied the potential association between CRP level higher than 40 mg/L and bacterial co-infection or efficacy of non-anti-MP antibiotics in 675 pediatric RMPP patients. We retrospectively analyzed the type and level of non-anti-MP antibiotics used and traditional bacterial culture of bronchoalveolar lavage fluid (BALF) in all the 675 patients. In addition, metagenomic analysis based next generation sequencing (NGS) were also performed with DNA extracted from BALF from 18 RMPP patients to verify the existence of bacterial co-infection. Our studies suggested that bacterial co-infection in the early stage of pediatric RMPP patients was low and CRP level might be mainly a marker of inflammation.

## Methods

### Study population

We retrospectively reviewed the medical records of 675 pediatric patients finally diagnosed with RMPP who were admitted in the Department of Respiratory Medicine II at Beijing Children's Hospital between January 2008 and December 2015.

All patients showed symptoms of pneumonia at admission, including persistent fever (>38.5°C), cough, and abnormal chest imaging. MP infection was diagnosed based on the demonstration of an immunoglobulin M-specific anti-MP antibody titer ≥ 1:320 or four-fold rising titer in acute and convalescent serum specimens. RMPP was diagnosed according to the following criteria: (1) patients with MPP had persistent fever and deterioration of clinical and radiological findings after administration of macrolide antibiotics for 7 days or more (1, 14, 15); (2) peripheral blood CRP were higher than 40 mg/L, an useful indicator of RMPP (3). All the 675 patients received bronchoscopy lavage therapy twice within 15 days in our hospital, one at day 6 to day 10 of the disease course and the other one at day 11 to day 15. Patients with respiratory virus infection, asthma, immunodeficiency, other chronic diseases, or with disease courses of more than 10 days before admission, or patients refused bronchoscopy lavage therapy (BLT) or received BLT at other hospitals or received BLT once in our hospital, were excluded from current study.

All patients were previously treated for at least 2 days (anti-MP or non-anti-MP antibiotics or glucocorticoid singly or in combination) at other hospitals or our outpatient clinic before admission to our inpatient department. Within the first 15 days of disease course: patient information, including age and gender, was collected; Blood tests for white blood cells (WBC) and CRP were performed; Treatment with non-anti-MP antibiotics were administrated; And the result of conventional bacterial culture and NGS of BALF, patient response, length of hospital stay, and complications mainly bronchitis obliterans (BO) were recorded.

**Figure 1:**
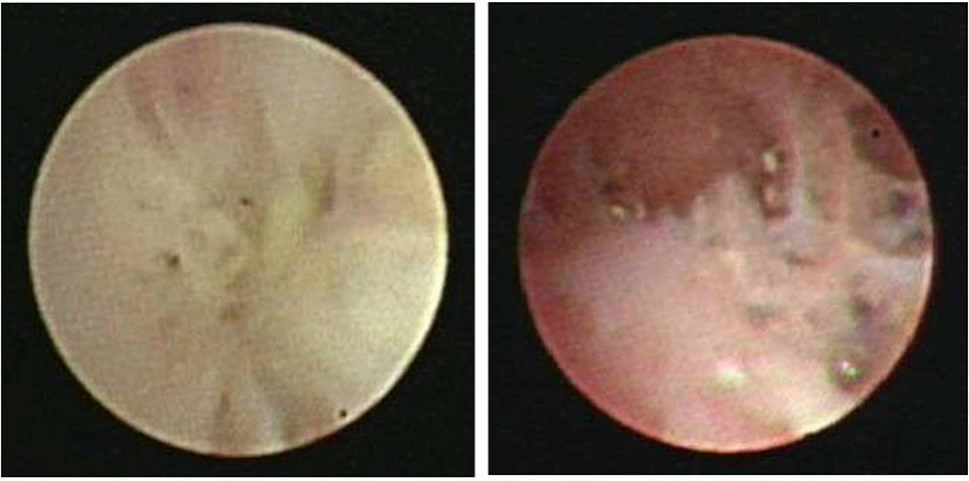
Fiberoptic bronchoscopy revealed the obliteration of the lumen of bronchial branches, bronchial segments, or bronchial subsegments.

BO is the most important complication in RMPP patients. The diagnosis of BO was based on the obliteration of the lumen of bronchi, bronchial branches, bronchial segments, or bronchial subsegments under bronchoscopy, which were evaluated by at least two clinicians (Figure 1).

### Fiberoptic bronchoscopy and BALF collection

BALF samples were collected using flexible fiberoptic bronchoscopy. Briefly, after working tip of bronchoscope was inserted into damaged lung lobe, warm sterile saline was instilled and recovered with gentle suction. 1.5–2 mL of BALF from each patient were used for traditional bacterial culture within half an hour of collection. Another 1.5–2 mL of BALF samples from 18 patients were used for meta-genomic sequencing.

### Bacterial culture of BALF samples

Each BALF sample was inoculated onto a Columbia blood agar plate, a vancomycin-containing chocolate agar plate, and a crystal violet-containing MacConkey agar plate, which were cultured at 37°C for two days. Bacterial strains in the cultures were screened using MALDI-TOF MS (bioMérieux, France) or Vitek 2 system (bioMérieux, France). Antimicrobial susceptibility testing was performed using a disc diffusion method or a broth microdilution method with a Phoenix100 automated microbiology analyzer (BD Research Inc., USA) or a Vitek 2 system (bioMérieux, France) according to CLSI standards.

### NGS procedure for BALF

In 2015, meta-genomic sequencing using NGS technology was available in our hospital. Therefore, BALF samples from 18 patients whose parents agreed with NGS detection and who hadn't received non-anti-MP antibiotics when they were collected at day 6 to day 10 of their disease courses were subject to NGS analysis. Specifically, half milliliter of BALF and 1 g of 0.5 mm glass beads were mixed vigorously at 2800-3200 RPM for 30 min in a 1.5 mL microcentrifuge tube. After spinning down, 0.3 mL of supernatants were transferred into a new 1.5 mL microcentrifuge tube for DNA extraction using a TIANamp Micro DNA kit (DP316, TIANGEN BIOTECH). DNA libraries were constructed through DNA-fragmentation, end-repair, adapter-ligation and PCR amplification. The quality of constructed DNA libraries was examined using an Agilent 2100 Bioanalyzer (Agilent, United States). Sequencing was performed using a BGISEQ-100 platform (BGI,Shenzhen China).

Sequencing data were cleaned up by removing low-quality or short reads (length < 35bp), followed by computational subtraction of host human sequences (hg19) using Burrows-Wheeler Alignment (BWA:http://bio-bwa.sourceforge.net/). The resulting clean sequencing data were mapped to four microbial genome databases for viruses, bacteria, fungi and parasites, which were downloaded from NCBI (ftp://ftp.ncbi.nlm.nih.gov/genomes/). RefSeq contains 2,700 whole genome sequences of viral taxa, 1,494 bacterial genomes or scaffolds, 73 fungi related to human infection, and 47 parasites associated with human diseases.

### Clinical outcomes

The body temperature and CRP value were reevaluated at 5 days and 4-6 days after treatment, respectively. Patients' response to therapy was classified into either clinical improvement or clinical failure. Clinical improvement was defined as resolution of fever along with declining of CRP level (at least 1/3 decrease of the basal level). Clinical failure was defined as either persistence of fever or no declining of CRP level (less than 1/3 decrease of the basal level or increase). Relapse was defined as increase of body temperature after it declined to normal level for at least 24 h during or after treatment period. No relapse was defined as body temperature declining to and staying at normal level for at least 5 days during or after treatment periods.

### Statistical analyses

SPSSversion 13.0 (SPSS Inc, Chicago, IL) was used for statistical analyses. All statistical hypothesis tests were 2-sided, and *P* < 0.05 were considered statistically significant.

### Ethics statement

This study was approved by the Ethics Committee of Beijing Children's Hospital. Written informed consent was obtained from the parents or the guardians of participants. In accordance with the Declaration of Helsinki and the local ethics committee requirements, the bronchoalveolar lavage fluid collection protocol in this research was based on the following standards:(1) Potential risk of the minor discomfort was minimized by utilizing only well trained personnel for the procedures; (2) Written informed consent was provided to the parents or the guardians of children; (3) The participants and their parents or their guardians had the right to decide whether or not to participate; (4) The participants in this study will not receive direct benefits from the proposed research. However, the benefits to society could be critical to guide rational therapy and prevent antibiotic misuse or overuse.

## Results

### Clinical characteristics of patients

**Figure 2:**
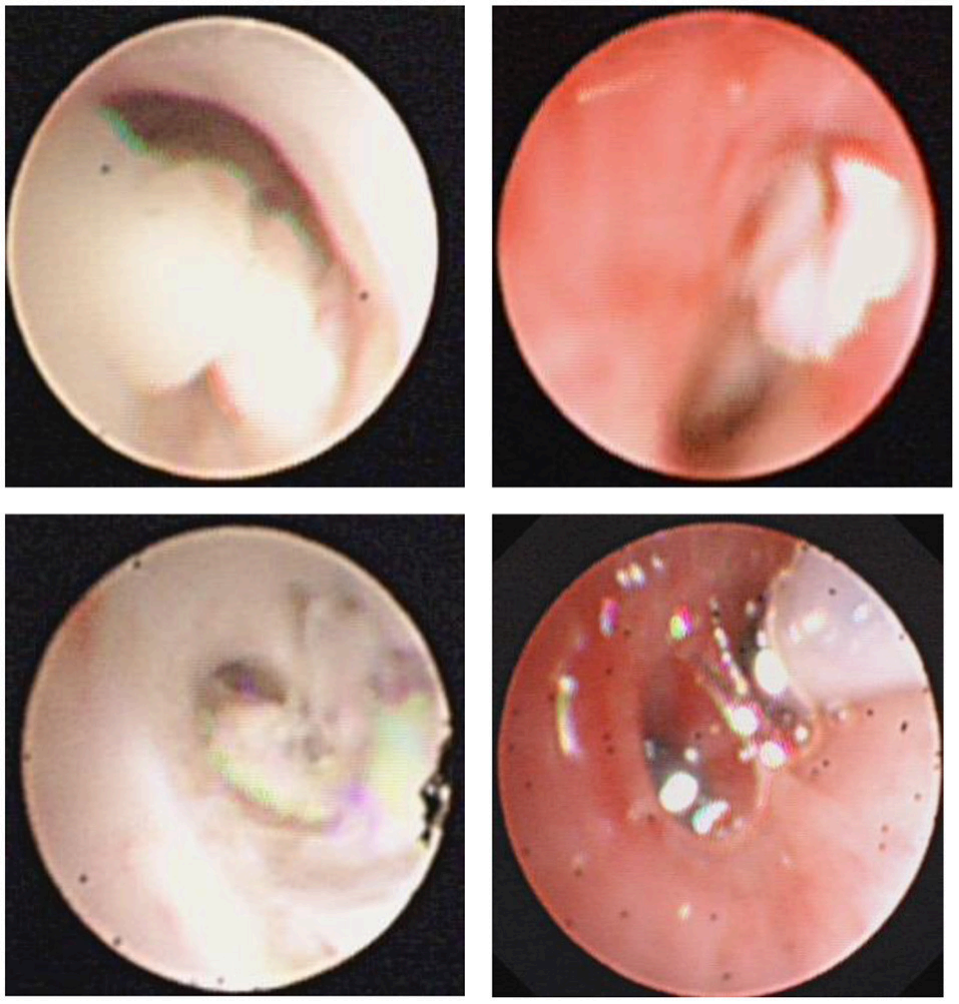
Fiberoptic bronchoscopy revealed mucous plug which suggested airway hypersecretion or airway obstruction in RMPP patients.

**Figure 3:**
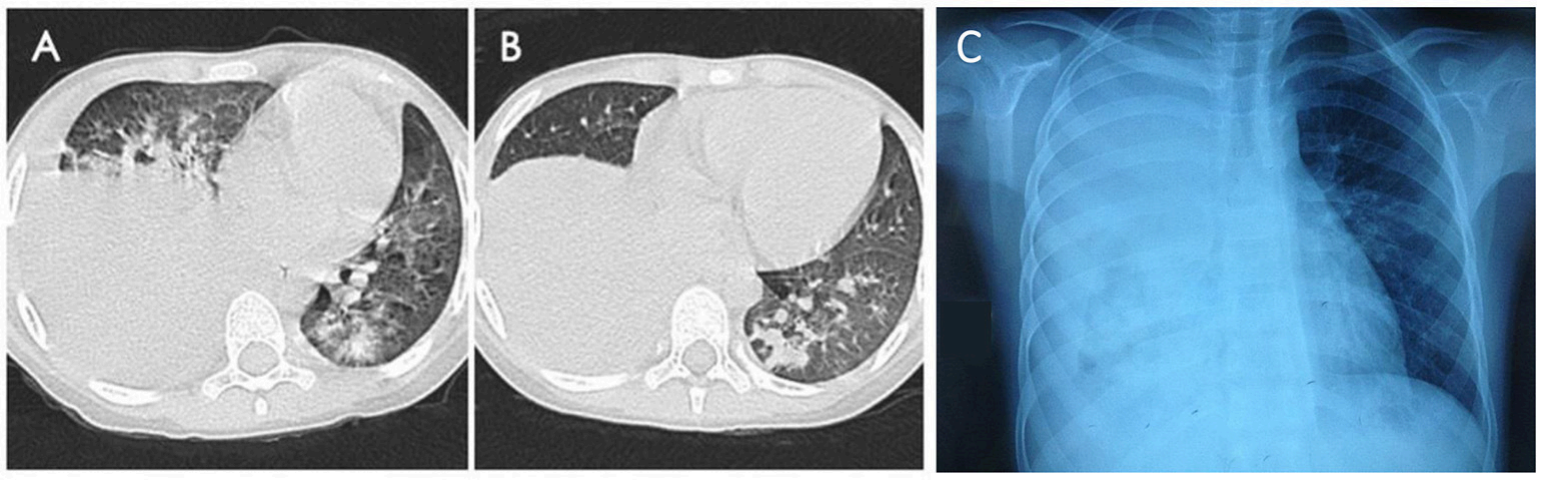
Chest imaging revealed high-density consolidation in right lung **(A,B)** with right pleural effusion **(C)** in RMPP patients.

**Figure 4:**
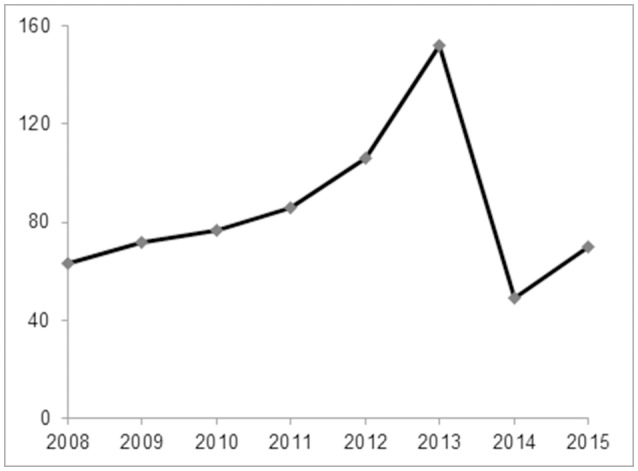
The annual case number of RMPP between January 2008 and December 2015. The number of patients enrolled in 2013 (152 cases) was the most followed by 2012 (106 cases), while only 49 patients were enrolled in 2014, which coincided with a RMPP outbreak around 2012-2013.

A total of 675 childhood RMPP patients (from 3 years 4 months to 15 years 8 months, mean age 7 years 11 months) were enrolled in our study. 351/675 patients (52%) were male and 324/675 (48%) were female. The patients had typical symptoms related to inflammation and infection such as airway hypersecretion in bronchoscopy (Figure 2) and high-density consolidation in chest imaging (Figure 3). As shown in Figure 4, the number of patients enrolled in 2013 (152 cases) was the most followed by 2012 (106 cases), while only 49 patients were enrolled in 2014, which coincided with a RMPP outbreak around 2012-2013. The averaged WBC counts (the maximal WBC count within the first 15 days of disease course of each patient was used for calculation of the mean) of all patients were (10.28 ± 3.56) × 10^9^ /L.

### Bacterial co-infection was rare in pediatric RMPP patients

Traditional bacterial culture of first BALF samples revealed 18 cases (case number 1-18, 18/675, 2.67%) positive for bacterial strains, including 10 cases (10/18, 55.56%) for *Streptococcus pneumoniae*, 3 cases (3/18, 16.67%) for *Staphylococcus aureus*, 3 cases (3/18, 16.67%) for *Haemophilus influenzae*, and 2 cases (2/18,11.11%) for *Moraxella catarrhalis* (Table 1), suggesting that bacterial co-infection occurred rarely in pediatric RMPP patients. We also found that the bacterial culture with the second batch of BALF samples identified less cases with co-infection as compared to that with the first batch of BALF samples (6/675, 0.89%) (Table 1). All cases positive for bacterial co-infection identified by the second batch of BALF culture were among the cases identified by the first batch of BALF culture (Table 1). The WBC counts were elevated in 6 cases (10.6~16.8 × 10^9^ /L).

**Table 1:** Culture results and clinical characteristics of 18 culture-positive RMPP patients.

	First BALF sample^*^				Second BALF sample^*^				**WBC (× 10^9^/L)**	**CRP (mg/L)**	**Non-anti-MP antibiotics**
**Case number**	**Sp**	**Sa**	**Hi**	**Mc**	**Sp**	**Sa**	**Hi**	**Mc**			
Case 1	+				+				7.7	58	A
Case 2	+				+				8.9	72	A
Case 3	+				+				13.1	57	B
Case 4		+							5.9	75	A
Case 5		+							9.9	65	B
Case 6			+						7.3	58	A
Case 7				+					16.8	78	A
Case 8				+					9.5	69	A
Case 9	+								6.3	94	B
Case 10	+				+				8.5	98	B
Case 11	+								9.3	102	B
Case 12	+								9.8	104	B
Case 13	+								16.7	106	D
Case 14			+				+		9.2	115	A
Case 15	+				+				15.3	125	D
Case 16		+							10.6	144	A
Case 17			+						15	127	A
Case 18	+								12.8	209	D

* *Culture results for the first/second BALF sample. Short names: Sp, Streptococcus pneumoniae; Sa, Staphylococcus aureus; Hi, Haemophilus influenzae; Mc, Moraxella catarrhali*.

### Non-anti-MP antibiotics were misused and overused in pediatric RMPP patients

All the patients were treated with anti-MP antibiotics and bronchoscopy lavage therapy along with other basic therapies, such as glucocorticoid according to the guidelines of the Chinese Medical Association (14, 15). The mostly administrated anti-MP antibiotic was macrolide followed by ciprofloxacin and doxymycin.

The overall rate of non-anti-MP antibiotics usage in all the 675 patients was 93.33% (630/675). When classifying the non-anti-MP antibiotics used in this study based on drug generations and types: second-generation non-anti-MP antibiotics cephalosporins and amoxicillin as Group A, third-generation cephalosporins as Group B, fourth-generation cephalosporins as Group C, teicoplanin, vancomycin, and linezolid as Group D, ertapenem, meropenem, and imipenem as Group E (If more than one non-anti-MP antibiotics were sequentially used for a patient, the higher generation of antibiotics was recorded), we found that about 80% of the patients were treated with generation two to four non-anti-MP antibiotics (group A: 324/675, 48%; group B: 264/675, 39.11%; group C: 12/675, 1.78%). Worth to note that about 10% patients were treated with last line antibiotics in group D (54/675, 8.00%) or group E (12/675, 1.78%). Moreover, 36 patients (36/675, 5.33%) were treated with a combination of two non-anti-MP antibiotics. Among the patients in different CRP groups 40 mg/L < CRP ≤ 80 mg/L, 80 mg/L < CRP ≤ 120 mg/L, 120 mg/L < CRP ≤ 160 mg/L and CRP > 160 mg/L, 94.90% (279/294), 87.57% (155/177), 92.66% (101/109), and 100% (95/95) were treated by non-anti-MP antibiotics along with anti-MP antibiotics (Table 2), respectively, suggesting that clinicians tended to prescribe non-anti-MP antibiotics to patients with high CRP.

**Table 2:** Laboratory examination, bacterial culture results of BALF, and Non-anti-MP antibiotic usage of RMPP patients.

	**Patients(40 < CRP ≤ 80 mg/L)**	**Patients(80 < CRP ≤ 120 mg/L)**	**Patients(120 < CRP ≤ 160 mg/L)**	**Patients(CRP > 160 mg/L)**	**Total patients**
Case number (%)	294 (43.6%)	177 (26.2%)	109 (16.1%)	95 (14.1%)	675
Average WBC (× 10^9^/L)	8.69 ± 3.49	10.19 ± 4.04	10.45 ± 4.08	11.14 ± 4.40	10.28 ± 3.56
Number of Positive bacterial culture of first BALF (%)	8 (2.72%)	6 (3.39%)	3 (2.75%)	1 (1.05%)	18 (2.67%)
Antibiotic A(cases)	174	76	47	27	324
Antibiotic B(cases)	96	71	43	54	264
Antibiotic C(cases)	2	2	4	4	12
Antibiotic D(cases)	7	5	11	31	54
Antibiotic E(cases)	0	3	3	6	12
Combined usage of 2 groups of antibiotics(cases)	0	2	7	27	36
Without non-anti-MP antibiotics(cases)	15	22	8	0	45
Use of non-anti-MP antibiotics (%)	279 (94.9%)	155 (87.6%)	101 (92.7%)	95 (100%)	630 (93.3%)

*Based on their CRP levels, we divided the patients into four groups: 40 mg/L < CRP ≤ 80 mg/L; 80 mg/L < CRP ≤ 120 mg/L; 120 mg/L < CRP ≤ 160 mg/L; CRP > 160 mg/L*.

### NGS could be a useful tool for detecting bacterial co-infection in pediatric RMPP patients

NGS was performed in 18 cases (case number 19-36, Table 3). We analyzed the number of unique reads, which could be aligned to pathogen's genomic sequences, and the coverage of pathogen genome aligned by the unique reads. NGS results revealed that MP was the dominant pathogen in the patients' BALF as evidenced by both large number of unique reads (above 700) and high level of coverage (over 10%) in every case. Non-MP bacteria were detected in six cases (Table 1), but with only very few unique reads (< 3 and) and poor coverages (~ 1%) (Table 1). Moreover, the bacteria detected by NGS were not commonly see. Together, we believed that the NGS results for these six cases were false positive, which was confirmed by our bacterial culture results of the first and second BALF, both of which were negative in these 18 cases. Moreover, NGS results didn't show infection by fungi, parasites or viruses.

**Table 3:** Results of Next-generation sequencing in 18 culture-negtive RMPP patients.

**Case number**	***Mycoplasma pneumoniae***		**Other bacteria**			**WBC(× 10^9^/L)**	**CRP(mg/L)**	**Non-anti-MP antibiotics**
	**Unique reads**	**Coverage rate (%)**	**Species**	**Unique reads**	**Coverage rate (%)**			
19	895	15		0		4.51	71	No
20	1,898	27	*Prevotella melaninogenica*	2	0.0084	14.94	78	No
21	6,226	36.8		0		16.26	80	No
22	45,352	96		0		9.88	86	No
23	12,350	68.3		0		10.85	93	No
24	5,660	35.8	*Streptococcus oralis*	1	0.0113	12.65	99	No
25	8,970	54.2		0		13.43	123	A
26	973	15.3	*Rothia mucilaginosa*	1	0.0078	8.96	128	A
27	5,783	38.1		0		6.74	131	A
28	4,728	21.9		0		11.69	136	A
29	27,621	79.2	*Propionibacterium acnes*	1	0.0065	5.97	139	A
30	7,835	51.8		0		15.61	141	A
31	5,733	35.3		0		7.3	166	A
32	37,957	94.8		0		8.9	168	B
33	9,860	57.3		0		10.9	170	B
34	3,560	30.2		0		15.43	174	B
35	46,398	95	*Propionibacterium acnes*	2	0.0096	20.15	213	B
36	721	10.1	*Methylobacterium radiotolerans*	2	0.0108	17.31	354	B

*NGS results revealed that MP was the dominant pathogen in the patients' BALF as evidenced by both large number of unique reads (above 700) and high level of coverage (over 10%) in every case. Non-MP bacteria were detected in case 2,6,8,11,17, and 18, but with only very few unique reads (< 3 and) and poor coverages (~ 1%)*.

### Clinical outcomes were not improved with administration of non-anti-MP antibiotics in pediatric RMPP patients

**Figure 5:**
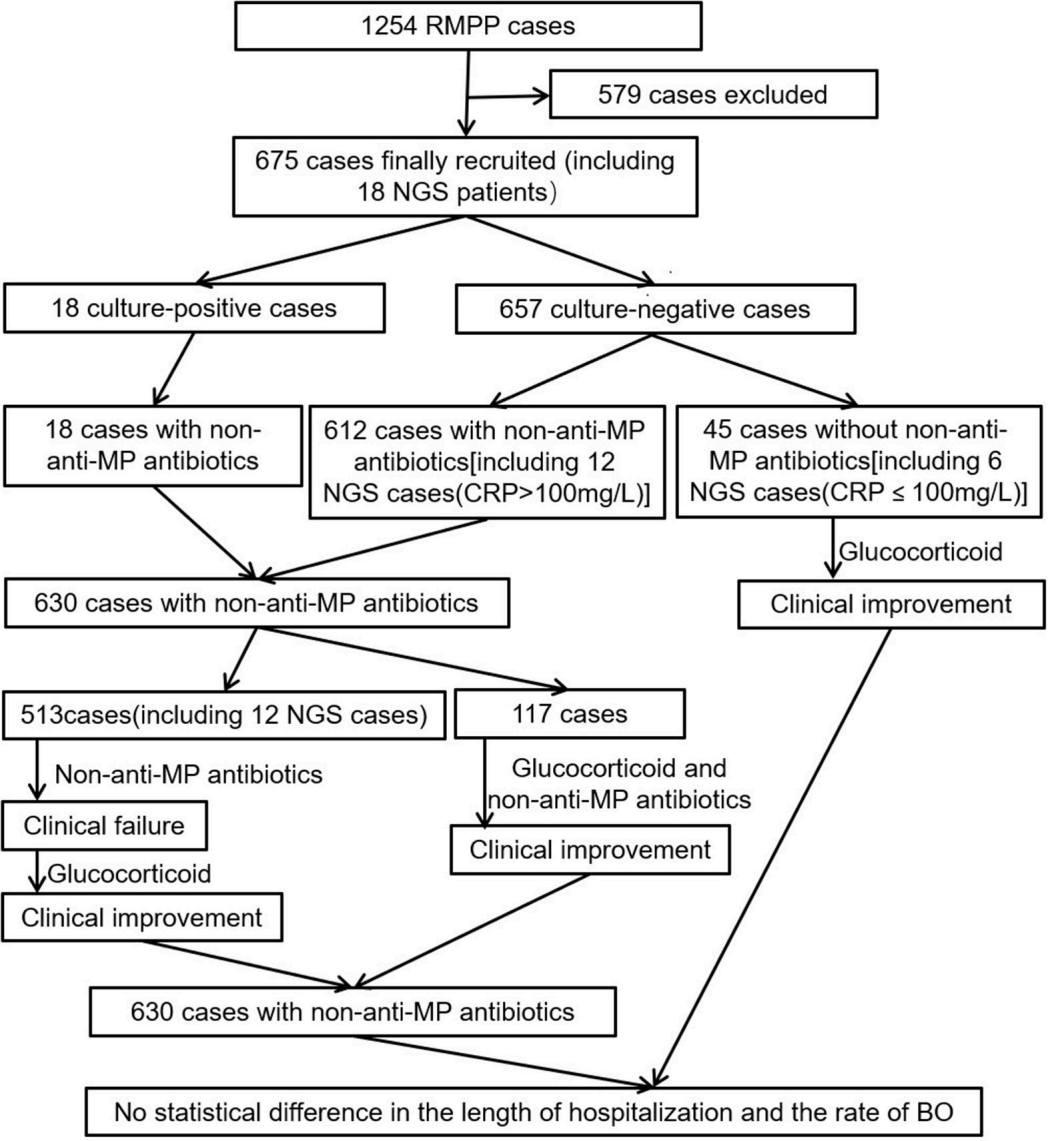
Flow diagram of patient enrollment and treatment.

First of all, we observed that in the 45 patients (including 6 NGS cases whose CRP were lower than 100 mg/L, Table 3, Figure 5) without non-anti-MP antibiotics treatment, their body temperature dropped to normal within 48 h after being treated with glucocorticoid. All of these patients achieved clinical improvement without relapse, which suggested that glucocorticoid was effective in treating pediatric RMPP patients.

Secondly, 501 patients were administrated of non-anti-MP antibiotics for at least 5 days before glucocorticoid therapy (Figure 5). We observed that administration of non-anti-MP antibiotics was ineffective in regard of patients' body temperature control, which led to clinical failure. After administration of glucocorticoid, the body temperature of these patients dropped to normal within 96 h and clinical improvements were achieved with no relapse, which further suggested that glucocorticoid, but not non-anti-MP antibiotics, was effective in treating pediatric RMPP patients.

However 117 patients achieved clinical improvement without relapse after being treated with non-anti-MP antibiotics and glucocorticoid within 2 days (Figure 5). We couldn't know whether non-anti-MP antibiotics or glucocorticoid singly were effective.

To improve our clinical practice and reduce the misuse and overuse of non-anti-MP antibiotics, we used NGS analysis to quickly detect whether a pediatric RMPP patient had non-MP co-infection and directed our clinical practice of administration of non-anti-MP antibiotics. Patients whose CRP were higher than 100 mg/L were usually immediately administrated of non-anti-MP antibiotics because of their sharply increased CRP levels. We performed NGS analysis on 12 patients (CRP higher than 100 mg/L) who were administrated of generation two or three non-anti-MP antibiotics from Group A or Group B once the samples for NGS examination were collected after admission to our department (Table 3). The NGS results came back within 72 h and revealed no bacterial co-infection in all cases (Table 1). We promptly discontinued the non-anti-MP antibiotics and switched to glucocorticoid therapy in these cases. All these patients achieved clinical improvement without relapse.

The length of hospitalization was 12.07 ± 2.597 days in patients without non-anti-MP antibiotics and 12.32 ± 2.731 days in patients with non-anti-MP antibiotics (*P* = 0.553). All patients had been followed at least 3 months. The rate of BO was 4.4% (2/45) in patients without non-anti-MP antibiotics and 5.6% (35/630) in patients with non-anti-MP antibiotics (4.4 vs. 5.6%; *P* = 1.000). There was no statistical difference in the length of hospitalization and the rate of BO between two groups of patients, which suggested the use of non-anti-MP antibiotics couldn't improve the clinical outcomes. Furthermore, there were no deaths in our study.

## Discussion

Antibiotic resistance is a pressing global health problem and has become a major public-health concern (16). Bacterial resistance to antibiotics, both at individual and community levels, is an unavoidable side-effect of consumption of these drugs (17). Due to misuse and/or overuse of antibiotics in clinic, more and more drug-resistant bacteria have emerged recently, some of which even resist to multiple or most of currently available antibiotics, thus being called super bacteria. Those super bacteria make it difficult to cure the diseases related to bacterial infection (17, 18). In addition, macrolide resistance rates of MP are very high in Asia especially in China, and high up to 97.0% in the Beijing population in 2012 (17, 18). Therefore, it is extremely important to control the application of antibiotics in clinic to minimize the occurrence of antibiotic resistance (16–18).

MPP is a community-acquired pneumonia occurring primarily in healthy children. It has been confirmed that excessive immune response of the host in RMPP plays an important role in the development of pulmonary lesions (19–24). Therefore, glucocorticoid is very important in the treatment of RMPP (1, 2, 4). Many studies showed that glucocorticoid could significantly improve the clinical outcomes of children with RMPP (1, 2, 5, 25, 26), which was also confirmed in this study.

High level of CRP is another prominent feature of RMPP (1–6, 25). CRP is a marker of the acute phase of RMPP and synthesized mainly by hepatocyte. In addition, it can be produced by alveolar macrophages and may be involved in pulmonary immune response (27, 28). In response to inflammation, cell damage, or tissue injury, plasma CRP level can rapidly and dramatically increase, which has been used as a marker to monitor infections and many destructive/inflammatory conditions. In general, a higher CRP level may indicate a more severe bacterial infection or stronger immune response. However, it is unclear whether the high level of CRP in RMPP is only caused by excessive immune response or by a combination of excessive immune response and bacterial co-infection, the answers of which will be extremely important to guide clinical application of antibiotics and glucocorticoid.

Currently, only a few papers talked about bacterial co-infection mainly in MPP not RMPP patients, and these studies didn't concern about the use of antibiotics. Song reported that 2% (173/8612) of MPP patients had bacterial co-infection, which was consistent with our results (29). In Chen's study, 10.9% (22/201) of MPP patients had bacterial co-infection (30), which was much higher than our results and might be due to its small sample size. In Izumikawa's study, non-MP bacteria were detected in 5.7% (2/35) of fulminant MPP patients but not considered as causative agents (31). In You's study, none of RMPP patients (0/12) had bacterial co-infection (32). In our current study, we saw 2.67% of total patients had positive bacterial cultures of first BALF (Table 1), and only 0.89% of the patients had positive bacterial cultures of second BALF, which also suggested low infection rates of bacteria other than MP in pediatric RMPP patients.

Due to limitation, traditional bacterial culture could not grow all bacteria in BALF. Previous study (33, 34) showed molecular detection of pathogens in BALF samples was a powerful tool in lower respiratory tract infection patients, particularly in patients treated with antibiotics. Yamasaki and colleagues utilized clone library analysis of 16S rRNA to analyze BALF organisms in community-acquired pneumonia (CAP) patients and detected many bacteria which could not grow in traditional bacterial culture (35), suggesting that oral/anaerobe agents may have some pathogenic role in CAP. In our study, we used NGS meta-genomic analysis, a very sensitive molecular method to confirm bacterial co-infection (36, 37). Our NGS results showed that MP was the predominant phylotype in our RMPP patients, which was consistent with 11 MPP cases in Yamasaki's study (35). We also detected few oral/anaerobe agents by NGS, but according to the few unique reads (< 3) of these agents and large number of unique reads (>720) of MP, the percentages of these agents (<0.3%) in our study were much lower than those in Yamasaki's study. The possible explanation for the difference is that our patients were children younger than 16 years old without underlying diseases, while the MPP patients in Yamasaki's study were aged 16 to 45 years with at least one comorbid illness. The oral/anaerobe agents in our sample could have been contaminants due to high sensitivity of NGS method and low detecting percentages of these agents. Therefore, both traditional bacterial culture and NGS analysis revealed a low rate of bacterial co-infection in children with RMPP, indicating that CRP increase in RMPP might mainly result from inflammation.

Most RMPP cases were reported in East Asia and we found although there was no evidence of bacterial co-infection in previous studies, surprisingly, non-anti-MP antibiotics were commonly administrated to pediatric RMPP patients not only in East Asia (1, 2, 31, 38–41), but also in Australia (42) and USA (43–45). In the present study, up to 93.33% of total patients were treated with non-anti-MP antibiotics, even last-line antibiotics, such as glycopeptides or carbapenems (Table 2). Moreover, 5.33% of patients simultaneously received two kinds of non-anti-MP antibiotics. Most patients in our study came from all over the country and had been combined with non-anti-MP antibiotics treatment at their local hospitals. However, we found that the addition of non-anti-MP antibiotics didn't significantly improve the clinical outcomes of childhood patients with RMPP. Firstly, most patients (558/675, 82.7%) including ones without non-anti-MP antibiotics treatment in our study showed clinical improvement during glucocorticoid treatment, while 501 patients initially using non-anti-MP antibiotics showed clinical failure without glucocorticoid treatment (Figure 5). Thus, the clinical improvement might be mainly due to glucocorticoid treatment but not non-anti-MP antibiotics. Secondly, all patients whose CRP were higher than 100 mg/L with NGS results received Group A or B non-anti-MP antibiotics only for 2–3 days, which were promptly discontinued once NGS result didn't reveal any bacterial co-infection (Table 3, Figure 5). We did not found any relapse after discontinuation of non-anti-MP antibiotics treatment. Thirdly, there was no statistical difference in the length of hospitalization and the rate of BO between patients with and without non-anti-MP antibiotics. These results suggested that there might not be bacterial co-infection in these patients and non-anti-MP antibiotics treatment was not necessary.

In general, WBC in peripheral blood increases in case of bacterial infections, which could be another indicator of bacterial co-infection. In present study, WBC in most RMPP patients was normal or mildly elevated, which also suggested that CRP increase might only relate to excessive immune response in RMPP. Some RMPP patients might have elevated WBC, which might indicate potential bacterial co-infection. In our NGS study with 18 patients (Table 3), five patients had obvious elevated WBC in periphery blood (even up to 20.15 × 10^9^/L), but did not have bacterial co-infection as shown by our NGS analysis and bacterial culture of BALF. Therefore, elevated WBC and CRP in RMPP patients might not be indicators of bacterial co-infection.

*S. pneumoniae* (10 patients) were the most commonly identified bacteria in our 18 patients with positive bacterial culture of first BALF. Except that, these patients demonstrated normal or only mildly elevated WBC, and mucus plug seeing with bronchoscopy which were almost identical to RMPP patients without bacterial pneumonia. These results suggested that the detected bacteria might be either only some colonized bacteria in the airways instead of the ones responsible for invasive infections or only responsible for minor infectiveness. In addition, six culture-positive patients had the same bacteria detected in the first BALF and the second BALF samples, and these patients had clinical improvement although they were still culture-positive after treatment of non-anti-MP antibiotics, indicating the detected bacteria might have colonized in airways.

Our study has several limitations. The sample size for NGS analysis was small due to cost issue. Serum procalcitonin wasn't analyzed due to its low specificity in our hospital [moreover Çolak A reported that CRP might be a more valuable marker than procalcitonin in patients with lower respiratory tract infections (46)]. Additionally, non-anti-MP antibiotics in most patients had been used for at least 2 days before the first BALF was collected for bacterial culture, which might lead to the low positive rate for bacteria culture.

Our study confirms that bacterial co-infection in pediatric RMPP is rare. In our opinion, although their pulmonary injury are severe, the general conditions of most RMPP patients were good. Therefore, we shouldn't routinely combine non-anti-MP antibiotics with anti-MP antibiotics for pediatric RMPP patients except those critical MPP patients in order to reduce the chance of antibiotic resistance and economic burden of patients. NGS would be a great tool in helping to quickly detect bacterial co-infection and direct antibiotic application to RMPP patients.

## Author contributions

J-RL and JL conducted the analysis, drafted and revised the initial manuscript, and J-RL is the guarantor of the article's findings. FD, H-ML, HL, X-LT and Y-LG advised on the design of the analysis and revised the manuscript. S-YZ conducted the analysis, revised the initial manuscript. All authors discussed the results, commented on the manuscript, and agreed to be accountable for all aspects of the work in ensuring that questions related to the accuracy or integrity of any part of the work are appropriately investigated and resolved.

### Conflict of interest statement

The authors declare that the research was conducted in the absence of any commercial or financial relationships that could be construed as a potential conflict of interest.
